# Open-Source Clinical Machine Learning Models: Critical Appraisal of Feasibility, Advantages, and Challenges

**DOI:** 10.2196/33970

**Published:** 2022-04-11

**Authors:** Keerthi B Harish, W Nicholson Price, Yindalon Aphinyanaphongs

**Affiliations:** 1 Grossman School of Medicine New York University New York, NY United States; 2 Law School University of Michigan Ann Arbor, MI United States; 3 Centre for Advanced Studies In Biomedical Innovation Law University of Copenhagen Copenhagen Denmark

**Keywords:** machine learning, artificial intelligence, medical economics, health policy, healthcare innovation

## Abstract

Machine learning applications promise to augment clinical capabilities and at least 64 models have already been approved by the US Food and Drug Administration. These tools are developed, shared, and used in an environment in which regulations and market forces remain immature. An important consideration when evaluating this environment is the introduction of open-source solutions in which innovations are freely shared; such solutions have long been a facet of digital culture. We discuss the feasibility and implications of open-source machine learning in a health care infrastructure built upon proprietary information. The decreased cost of development as compared to drugs and devices, a longstanding culture of open-source products in other industries, and the beginnings of machine learning–friendly regulatory pathways together allow for the development and deployment of open-source machine learning models. Such tools have distinct advantages including enhanced product integrity, customizability, and lower cost, leading to increased access. However, significant questions regarding engineering concerns about implementation infrastructure and model safety, a lack of incentives from intellectual property protection, and nebulous liability rules significantly complicate the ability to develop such open-source models. Ultimately, the reconciliation of open-source machine learning and the proprietary information–driven health care environment requires that policymakers, regulators, and health care organizations actively craft a conducive market in which innovative developers will continue to both work and collaborate.

## Introduction

### Background

Machine learning (ML) is a subset of artificial intelligence (AI) that uses training on existing data to generate insights on novel data. ML applications can augment physicians’ ability to make evidence-based decisions by synthesizing and applying more data points into practice than can any one individual. Currently, at least 343 AI-enabled tools have been cleared or authorized by the US Food and Drug Administration (FDA) to assist with functions including reading radiographs, classifying ophthalmic imaging, and interpreting electrocardiograms [1]. However, these technologies are still relatively novel, with the potential for widespread use in the near future.

Commercial innovations in modern medicine have largely taken advantage of proprietary information. However, software has had a longstanding paradigm bifurcation into proprietary and open-source software. Open-source software differs from proprietary software in the accessibility of its underlying code. Unlike proprietary software, open-source tools make their underlying code accessible to users. The use of proprietary software in American health care systems generally exceeds the use of open-source software [2]. However, select examples, such as the United States Veterans Affairs’ open-source VistA system, have found success in clinics and facilities [3]. Open-source software solutions have also been successfully implemented in developing nations’ health care systems [3].

Open source looks to be an important part of the health care ML landscape. This entry has already begun; for instance, developers building clinically oriented models often share source code as part of distribution. Examples of such models include those indicated for patient risk stratification [4], cancer therapeutic selection [5], pneumothorax detection [6], and pneumonia classification [7]. Activity in this space currently exists outside of structured market, regulatory, or implementation frameworks. In this paper, we evaluate the consequences of and raise considerations for the development and distribution of open-source ML models in health care settings. We consider factors contributing to the feasibility of deployment, the advantages of open source, and challenges faced by those seeking to develop and distribute open-source models.

### Feasibility

A total of 3 factors contribute to the feasibility of deploying open-source deep learning models. First, developing deep learning models, as compared to other health care solutions, requires relatively little capital on the part of developers. The collection of data is often passive, taking place routinely during encounters and hospitalizations. Although the curation of data may take effort, resources expended in collecting information for deep learning models are far less than those required while collecting information for drug development. Likewise, proving efficacy through retrospective and prospective validation can occur in a randomized fashion in the background of standard clinical operations. Performance standards can be assessed without changing the course of care. ML models also do not require the design and execution of randomized controlled clinical trials, which cost on average US $20 million per trial for stage III drug candidates [8]. Thus, open-source models require fewer incentives to recoup development costs. Given the favorable risk profile of constructing and deploying deep learning tools, developers have less incentive to keep their algorithms secret.

Second, the concept of open-source products is already familiar to the technology and information technology (IT) industries. Commonly recognized examples include Linux, the Apache HTTP Server, and Mozilla Firefox [9]. The global market for open-source projects across sectors was almost US $9 billion in 2016 and is expected to rise, with North America having the largest share [10]. Even in health care, the development of open-source models is nothing new. Scoring systems such as the PORT (Patient Outcomes Research Team), APACHE II (Acute Physiology and Chronic Health Evaluation II), and the Charlson Comorbidity Index are all open source and freely available [11]. These are all relatively simple models that apply logistic regression or points-based systems. The advent of deep learning and other sophisticated models may be considered in the context of these simpler models. Cultural, organizational, and policy factors have contributed to the notoriously slow adoption of technology in clinical settings [12]. Having strong precedents for the widespread utilization of open-source tools may decrease the magnitude of this barrier.

The third and most uncertain factor is the regulatory landscape for the entry of tools into the market. Regulation of ML in health care involves its own complex set of issues [13,14]. Many models developed and deployed in-house are unlikely to face much regulatory oversight, for various reasons that are still developing. Models developed in-house and shared noncommercially for in situ modification and deployment may still receive relatively little scrutiny. However, even at the most intense end of the scale of regulatory scrutiny, ML models under the FDA’s jurisdiction are typically eligible for the 510(k) approval process, allowing for the approval of a device via proof of equivalency to another device [15]. Thus, a deep learning model that is equally performant to an existing product can gain expedited approval. As of January 2022, at least 90% of AI-enabled models gained approval via the 510(k) pathway [1]. Open-source developers can utilize this same process to release models into the market.

### Advantages

There are 4 primary advantages to the development and integration of open-source ML models. First, the transparent nature of open-source software can potentiate enhanced integrity and performance. Unlike proprietary software, for which only purchasers can run models, anybody who has access to available open-source code can assess the model’s performance [16]. These circumstances thus allow for validation by greater numbers of people and on greater numbers of data sets. For tools requiring FDA approval, open-source models must either undergo a process demonstrating safety and efficacy or, more feasibly, undergo a process establishing performance equivalency to a model already in existence [13]. However, these validation processes are dependent on the data used to test the models at the time of appraisal. The FDA does not yet have a neutral third-party data set to validate individual developers’ models. Given these regulatory shortcomings, models require rigorous postmarketing surveillance [17]. Current efforts to interrogate proprietary software often reveal performance issues well after the commercial software has been widely distributed and implemented [18,19]. As compared to proprietary software, open-source tools would enable greater ability to detect deficits such as poor generalizability, previously unaccounted biases, and model drift.

Second, open source allows for the customization of models for a hospital’s specific population. ML tools, like therapeutics, are developed on data sets of large cohorts but ultimately applied to individuals. Thus, safety and efficacy vary among individuals and between specific populations [20]. When applied to medical informatics, this phenomenon is known as the “curly braces problem”: implementing models in new settings degrades their performance [21]. Facilities and departments using open-source models can somewhat mitigate this problem by calibrating the model weighting to achieve optimal performance on their unique patient populations. A cancer center, for instance, may want image reading models calibrated slightly differently from an emergency department. The capability to adjust source code to deliver increasingly personalized care may increase safety and efficacy.

Third, low-cost open-source options may speed up the adoption of ML technology in clinical settings. Inherent to the notion of open-source models is the availability of their code. Due to the nature of their transparency, open-source tools have historically been lower in price compared to their proprietary counterparts [22]. Despite a projected US $6.6 billion investment by developers and investors [23], health care facilities have proven slow to adopt ML technologies [24]. Among other reasons, hesitancy by clinicians and administrators due to potential financial or value-based consequences of using such technology hold back its implementation [24,25]. Decreasing the financial risks of adoption may encourage operational experimentation, particularly for hospitals that are naïve to ML tools.

Fourth, low-cost open-source options may increase competition, influencing price and functionality. The emergence of open-source models is not likely to end the development of proprietary technology. From word processors to COVID-19 decision support algorithms, the uptake of open-source tools has occurred alongside proprietary tools [9,26]. However, proprietary models, often priced higher than open-source models, will have to compete with effective “generic” models. Facilities looking to use deep learning for any given use case may confront a combination of proprietary and open-source options. The very existence of comparable open-source models forces proprietary developers to increase functionality in return for the higher cost [21]. Results may include a smoother user interface, enhanced integration with existing health care IT systems, or augmented implementation guidance or maintenance.

### Challenges

The implementation of open-source deep learning models also faces 4 primary challenges. First, engineering issues impact the feasibility of development and maintenance. A model is one piece of a multicomponent production pipeline. The code and infrastructure around the model, known as ML operations (MLOps), are necessary to make the model production ready. For the most part, commercial services include MLOps services with the purchase of proprietary models. These services would not be included with the implementation of isolated open-source model code.

The monitoring of inputs, an important component of MLOps, ensures that the model works as intended. Changes in inputs can cause a model to produce unexpected outputs. For example, a recent electronic health record upgrade at New York University (NYU) Langone Health caused a monitoring system to flag changes in model inputs, and we were immediately able to flag the change and fix the input mappings. Alternatively, the underlying population or treatments may change. For example, NYU Langone Health researchers trained a model to predict 2-month mortality [27]. In prospective validation, the team identified a subgroup of patients with lung cancer who were unexpectedly surviving beyond 2 months. In the intervening time between model training and prospective validation, the FDA approved pembrolizumab (Keytruda) for clinical use. Patients treated with Keytruda were no longer at high risk of short-term death.

Second, increased accessibility to source code exposes models to manipulation, especially by adversarial machine learning. Adversarial machine learning techniques involve feeding models misleading data to produce faulty outputs. Researchers have used such techniques to deceive models processing multiple forms of media, including images and text [28]. Reports have described engineered attacks in which experts have been unable to distinguish between data from patients and manipulated data [29,30]. Adversarial attackers with access to model source code could release models deliberately designed to negatively impact patient care. Alternatively, because these models are open source, the attacks are transparent and thus mitigatable. Additionally, the monitoring infrastructure still exists and if done correctly, should immediately flag these attacks.

Third, the intellectual property and incentive landscape for open-source medical ML models is complex. Attempting to maintain exclusivity for models is contrary to the spirit of open-source sharing. Even if developers were to attempt to seek some intellectual property protection, patents provide relatively weak protection for models (and no protection at all for the data on which models are based), based in part on US Supreme Court decisions that expansively defined the set of abstract ideas and natural laws that cannot be patented [31]. Secrecy, the principal alternative to patents, is similarly incompatible with an open-source model, though a combination of secrecy and licensing does enable variants such as open-source products solely for noncommercial uses.

The lack of exclusivity-based supracompetitive pricing limits the incentives available for the development and validation of open-source models. This especially constrains the activities model developers would be willing to undertake; cheaper work, such as model development based on existing in-house data sets or in silico validation, is substantially easier to justify and support than more expansive and expensive work, such as prospective clinical trials to validate model performance or generalizability across contexts, that is necessary for the evidence-based adoption of an AI model [32].

Fourth and finally, developers of open-source medical ML models face complex possibilities around the question of liability, namely, whether a model developer can face liability when patients are harmed based on the use of an arguably faulty model. Fully expanding upon the possibilities of liability is outside the scope of this work, not least because courts have yet to clarify the doctrine. The frequent finding of liability for upstream open-source model developers seems relatively unlikely [33]. Among other things, courts have been reluctant to impose product liability on software developers because software is only disputably a product. Intervening actors, such as the health system implementing (and perhaps modifying) an open-source model and the health care provider caring for the patient, further complicate the causal chain and the assignment of liability. Finally, licensing terms that include indemnification for liability and the reassignment of liability by insurers both add complexity to the liability landscape. Suffice it to say that liability remains an area of uncertain concern but seems unlikely to deter a substantial amount of open-source model development and collaboration, as evidenced in part by the sharing already occurring. Still, the area is one that developers should likely continue to monitor.

### Conclusions

In this viewpoint, we have evaluated factors involved in the development and deployment of open-source ML models for use in clinical settings, considering feasibility, advantages, and challenges inherent to such a framework. The benefits of open-source technology are largely known and accepted within the technology community. The forces holding back the adoption of the proposed technology, however, lie in the lesser-known aspects of the intersection between the data sciences, clinical sciences, and health care policy. Questions surrounding regulation, liability, and market forces predominate concerns about furthering the development of tools in a manner that potentially limits the extent of profit margins.

Given these outstanding questions, we believe that policymaker interventions have a fundamental role in enabling developers. A pragmatic start would be to ensure model generalizability. An overarching concern in the applicability of open-source models is the ability to use models in different settings while trusting that performance will remain strong, especially given the MLOps factors noted above and the possibility of patient injury (and potential liability) that might result from improper translation. Demonstrating generalizability, however, is expensive and as noted, patents, since they are not available, do not create incentives for incurring that expense. Policymakers could both encourage generalizability testing and reduce attendant expenses by helping to develop a unified infrastructure to enable such testing before sharing. Such an infrastructure could involve routinely updated test data sets, mock settings, and challenge queries. Generalizability infrastructure would make it easier to develop responsible open-source models and could also reduce redundant infrastructure effort by those whose resources could be better spent developing and improving models. Although we do not take a strong view as to who could best design such an infrastructure, the FDA seems a logical contender.

More generally, the role of forward-thinking governance remains critical to the development and deployment of open-source models. The FDA has recently announced that it was considering changes to its standard approval process directed at establishing more appropriate regulation regarding ML programs [13]. Changes include a precertification pilot program where companies are approved before they develop and release models. This allows for the release of new versions without subsequent safety and efficacy trials. Similar innovations may be required in regulatory bodies, clinical facilities, and the law to provide guidance that supports a sector that intertwines proprietary and open-source models. In the meantime, developers may need to shoulder some of the risk of promoting innovations to improve patient care, including through the sharing of open-source models.

## Abbreviations

AI: artificial intelligence
APACHE II: Acute Physiology and Chronic Health Evaluation II
FDA: US Food and Drug Administration
IT: information technology
ML: machine learning
MLOps: machine learning operations
NYU: New York University
PORT: Patient Outcomes Research Team

## References

[1] Artificial intelligence and machine learning (AI/ML)-enabled medical devices. US Food and Drug Administration.

[2] Olaronke I, Soriyan A, Gambo I, Olaleke J (2013). Interoperability in healthcare: benefits, challenges and resolutions. Int J Innov Appl Stud.

[3] Karopka T, Schmuhl H, Demski H (2014). Free/libre open source software in health care: a review. Healthc Inform Res.

[4] Zeiberg D, Prahlad T, Nallamothu BK, Iwashyna TJ, Wiens J, Sjoding MW (2019). Machine learning for patient risk stratification for acute respiratory distress syndrome. PLoS One.

[5] Huang C, Mezencev R, McDonald JF, Vannberg F (2017). Open source machine-learning algorithms for the prediction of optimal cancer drug therapies. PLoS One.

[6] Kitamura G, Deible C (2020). Retraining an open-source pneumothorax detecting machine learning algorithm for improved performance to medical images. Clin Imaging.

[7] Wang L, Lin ZQ, Wong A (2020). COVID-Net: a tailored deep convolutional neural network design for detection of COVID-19 cases from chest X-ray images. Sci Rep.

[8] Sertkaya A, Birkenbach A, Berlind A, Eyraud J, Eastern Research Group Inc (2014). Examination of clinical trial costs and barriers for drug development. Office of the Assistant Secretary for Planning and Evaluation.

[9] Androutsellis-Theotokis S, Spinellis D, Kechagia M, Gousios G (2011). Open source software: a survey from 10,000 feet. Foundations and Trendsin Technology, Information and Operations Management.

[10] MarketsandMarkets Research.

[11] Aakre C, Dziadzko M, Keegan M, Herasevich V (2017). Automating clinical score calculation within the electronic health record. A feasibility assessment. Appl Clin Inform.

[12] Cresswell K, Sheikh A (2013). Organizational issues in the implementation and adoption of health information technology innovations: an interpretative review. Int J Med Inform.

[13] Artificial intelligence and machine learning in software as a medical device. US Food and Drug Administration.

[14] Minssen T, Gerke S, Aboy M, Price N, Cohen G (2020). Regulatory responses to medical machine learning. J Law Biosci.

[15] Premarket notification 510(k). US Food and Drug Administration.

[16] Đurković J, Vuković V, Raković L (2008). Open source approach in software development - advantages and disadvantages. Manag Inf Syst.

[17] Hwang TJ, Kesselheim AS, Vokinger KN (2019). Lifecycle regulation of artificial intelligence- and machine learning-based software devices in medicine. JAMA.

[18] Obermeyer Z, Powers B, Vogeli C, Mullainathan S (2019). Dissecting racial bias in an algorithm used to manage the health of populations. Science.

[19] Singh K, Valley TS, Tang S, Li BY, Kamran F, Sjoding MW, Wiens J, Otles E, Donnelly JP, Wei MY, McBride JP, Cao J, Penoza C, Ayanian JZ, Nallamothu BK (2021). Evaluating a widely implemented proprietary deterioration index model among hospitalized patients with COVID-19. Ann Am Thorac Soc.

[20] Eichler H, Abadie E, Breckenridge A, Flamion B, Gustafsson LL, Leufkens H, Rowland M, Schneider CK, Bloechl-Daum B (2011). Bridging the efficacy-effectiveness gap: a regulator's perspective on addressing variability of drug response. Nat Rev Drug Discov.

[21] Hripcsak G, Ludemann P, Pryor T, Wigertz OB, Clayton PD (1994). Rationale for the Arden Syntax. Comput Biomed Res.

[22] Riehle D (2007). The economic motivation of open source software: stakeholder perspectives. Computer.

[23] Collier M, Fu R, Yin L (2017). Artificial intelligence: healthcare’s new nervous system. Accenture plc.

[24] Singh RP, Hom GL, Abramoff MD, Campbell JP, Chiang MF, AAO Task Force on Artificial Intelligence (2020). Current challenges and barriers to real-world artificial intelligence adoption for the healthcare system, provider, and the patient. Transl Vis Sci Technol.

[25] He J, Baxter SL, Xu J, Xu J, Zhou X, Zhang K (2019). The practical implementation of artificial intelligence technologies in medicine. Nat Med.

[26] Pearce JM (2020). A review of open source ventilators for COVID-19 and future pandemics. F1000Res.

[27] Major VJ, Aphinyanaphongs Y (2020). Development, implementation, and prospective validation of a model to predict 60-day end-of-life in hospitalized adults upon admission at three sites. BMC Med Inform Decis Mak.

[28] Finlayson SG, Bowers JD, Ito J, Zittrain JL, Beam AL, Kohane IS (2019). Adversarial attacks on medical machine learning. Science.

[29] Han X, Hu Y, Foschini L, Chinitz L, Jankelson L, Ranganath R (2020). Deep learning models for electrocardiograms are susceptible to adversarial attack. Nat Med.

[30] Du-Harpur X, Arthurs C, Ganier C, Woolf R, Laftah Z, Lakhan M, Salam A, Wan B, Watt FM, Luscombe NM, Lynch MD (2021). Clinically relevant vulnerabilities of deep machine learning systems for skin cancer diagnosis. J Invest Dermatol.

[31] Price WN (2016). Big data, patents, and the future of medicine. Cardozo L Rev.

[32] Stern A, Price WN (2020). Regulatory oversight, causal inference, and safe and effective health care machine learning. Biostatistics.

[33] Reutiman JL (2012). Defective information: should information be a product subject to products liability claims. Cornell J Law Public Policy.

